# IgG4-Related Disease: Endocrine Involvement, Clinical Implications, and Management Strategies

**DOI:** 10.15190/d.2025.18

**Published:** 2025-12-31

**Authors:** Mohamed Ahmed Mohamed, Ahmad Hammoud, Hadil Maher Jaber, Leen Nasser Shaban, Leen Abu Shaqra, Lara Ahmad Nazzal, Dyala Essam Haddadin, Farah Essam Haddadin, Nader Mohammad Alaqtash, Leena ElNaim MohamedAli Ahmed, Avantika Sujith

**Affiliations:** ^1^Medeor hospital, Abu Dhabi, United Arab Emirates; ^2^Ilia State University, Tbilisi, Georgia; ^3^European University, Tbilisi, Georgia; ^4^University of Georgia, Tbilisi, Georgia

**Keywords:** Endocrine, hypophysitis, IgG4-related disease, thyroid, pituitary, pancreas.

## Abstract

IgG4-related disease is a systemic fibroinflammatory disorder characterized by its tumefactive lesions, elevated serum IgG4 levels, and unique histological findings such as lymphoplasmacytic infiltrates in a storiform pattern. It affects a wide variety of organ systems however, endocrinological manifestations remain uncommon and underdiagnosed due to nonspecific presentations. The thyroid and pituitary glands are the most commonly affected endocrinological organs, with the thyroid presenting as Riedel’s thyroiditis or IgG4-related thyroiditis, and the pituitary involvement as hypophysitis. Symptoms could arise either due to direct organ infiltration or a decrease in hormone production and release. Diagnosing IgG4-related disease depends on serological, radiological, and histopathological findings. While immunosuppressive and biological agents are used for more severe or refractory cases, corticosteroids are the mainstay of treatment considering the autoimmune etiology of this disease. Early recognition of endocrinological involvement in IgG4-related disease is imperative for treating and preventing eventual irreversible glandular damage, highlighting the need for increased clinical awareness and multidisciplinary approaches amongst physicians. This review focuses on the endocrinological manifestations, clinical implications, and management strategies of IgG4-related disease.

## SUMMARY:

1. Introduction
2. Pathophysiology of IgG4-related disease
3. IgG4-Related Hypophysitis
4. Thyroid Involvement
  1. Riedel’s thyroiditis
  2. Hashimoto’s thyroiditis
5. Pancreatic involvement
6. Diagnostic Workup in Suspected Endocrine IgG4-RD
7. Treatment Strategies and Endocrine Outcomes
8. Conclusion

## 1. Introduction

IgG4-related disease (IgG4-RD) comprises a wide range of immune mediated conditions. It is a multisystem fibroinflammatory disorder characterized by its specific morphological findings and tumefactive lesions^1^. These diseases are distinguished by elevated serum IgG4 levels as well as certain key histopathologic features. Tissue biopsies show dense lymphoplasmacytic infiltrate with IgG4 plasma cells organized in a storiform pattern, obliterative phlebitis and an eosinophilic infiltrate^2,3^. IgG4-RD most frequently affects the organs of the gastrointestinal tract including the pancreas and the salivary glands ^4,5^. IgG4-RD can affect a wide variety of organ systems such as the respiratory and the central nervous system, and there have been 40 different locations of the diseases reported^4^.

Endocrine manifestations and involvement in IgG4-RD aren’t very common, for instance a prospective cohort study of IgG4-RD patients by Chen et al. found that 38.5% of patients with IgG4-RD exhibited pancreatic involvement, 2.5 % of the patients reported thyroid enlargement^6^. The thyroid gland is the most commonly involved endocrine organ, thyroid involvement can manifest as Riedel’s thyroiditis, Hashimoto thyroiditis, the fibrous variant of Hashimoto thyroiditis and Grave’s disease are all thyroid diseases classified within the spectrum of IgG4-RD^7,8^.

**Figure 1:**
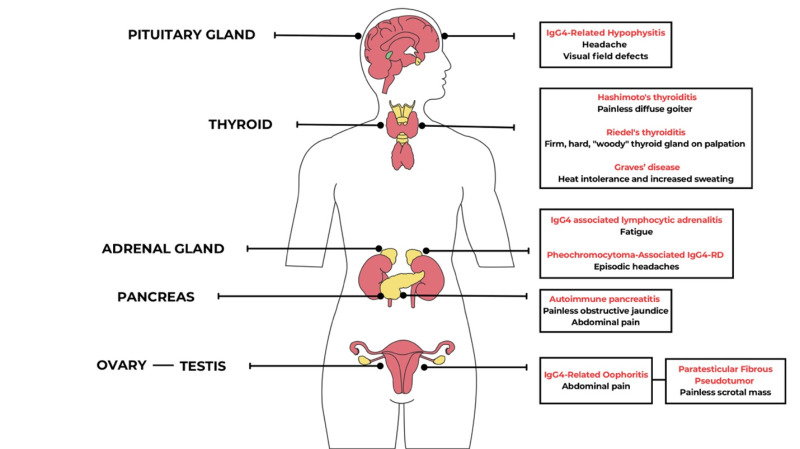
Illustration of the different endocrinological manifestations of IgG4-related disease in various organs

The pituitary gland is the second most commonly involved endocrine organ and manifests as IgG4-related hypophysitis^9^. Pancreatic involvement is also quite common in IgG4-RD. Type 1 autoimmune pancreatitis (AIP) is characterized by diffuse pancreatic swelling, irregular narrowing of the main pancreatic duct, painless jaundice and has histologic features of lymphoplasmacytic sclerosing pancreatitis^10,11^ (**Figure 1**).

The endocrine manifestations of IgG4-RD often share overlapping radiological and clinical features with other endocrine disorders increasing the risk of misdiagnosis, hence altering the therapeutic pathway to suboptimal strategies and despite their adverse effects (including affecting the endocrine system) corticosteroids remain the gold standard treatment, with limited data on potential interventions or adjunctive therapeutic options^12^.

This paper aims to comprehensively examine the association between IgG4 antibodies and endocrinopathies, with a gland focused analysis that also explores new therapeutic and diagnostic approaches to IgG4 conditions.

## **2.**Pathophysiology of IgG4-related disease

IgG4-RD is a result of chronic activation of the immune system and fibrosis of involved tissues, It is characterized by mass forming, tumor-like lesions that involve various tissues^13,14^. Histologically, these lesions are characterized by a dense lymphoplasmacytic infiltrates with numerous IgG4 plasma cells that are associated with storiform fibrosis, these findings are hallmarks of IgG4-RD^3^. These features suggest an antigen-driven immune response with T-cell activation that leads to cytokine release and B-cell activation with a switch to IgG4 production^15^.

The presence of numerous IgG4+ plasma cells in the affected tissues implicates B-cells as being the primary cell that drives the pathogenesis of IgG4-RD^15,16^. The involvement of B cells can be utilized therapeutically. Rituximab (a monoclonal antibody that targets CD-20, a B-cell surface marker) leads to B cell depletion, and has been used to induce remission in IgG4-RD^17,18^. Studies have shown that patients with inflammatory disorders possess increased peripheral plasmablast counts when compared to healthy patients ^16^. This finding is more pronounced in IgG4-RD, Wallace et al reported that plasmablast count can correlate with IgG4-RD activity, in addition plasmablast count dropped significantly with rituximab^16^

T cells also play a significant role in IgG4-RD as evidenced by CD4+ T cells presenting a substantial component of the mononuclear infiltrating cells found in the affected tissues^13,19^. The predominant subtypes of T cells involved included: Th2 cells, T follicular helper cells, CTL (Circulating Effector T cell), and T regulatory cell^15^. Cytokines released by Th2 cells (IL-4, IL-5, and IL-13) were found in high concentrations in both the tissues and blood of IgG4 patients^20-23^. Ectopic lymphoid structures (ELS) are frequently observed in IgG4-RD. ELS are classically defined as T and B cell aggregates with compartmentalized B cell and T cell-rich zones, development of follicular dendritic cell (FDC) networks and high endothelial venules in non-lymphoid target organs^14^.

CD4+CTLs are circulating effector CD4+ cells that express CD4+, CD27low and CD62low and have been described in IgG4-RD lesions. It has been shown that they secrete cytokines that are profibrotic such as TGF- β, IL-1b and IFN-γ in tissues of IgG4-RD patients^24^. They have been shown to decline following rituximab treatment suggesting that B and some T cells closely interact. It is believed that the proliferation of T regulatory cells (TREG) in the blood and tissues of IgG4-RD patients can also contribute to fibrosis via releasing TGF- β whereas In contrast, TGF- β release is frequently decreased in other autoimmune disorders^20,22,25^.

The exact function of IgG4 remains elusive and not explicitly understood^26,27^. The interaction between B lymphocytes, cytotoxic T lymphocytes, Th2 lymphocytes, TREGs and autoantibodies partially illustrate the pathogenesis of IgG4-RTD. Further studies are required to gain a better understanding of the pathways and how they can potentially be targeted to treat IgG-RTD.

## 3. IgG4-Related Hypophysitis

IgG4-related hypophysitis (IgG4-RH) is an autoimmune inflammation of the pituitary gland that along with hypertrophic pachymeningitis constitutes the most common IgG4-RD in the nervous system^26^. IgG4-RH can be identified secondary to a non-endocrine IgG4-RD manifestation in 1.7-8% of the cases or as an isolated condition without multiorgan involvement^28^.

Due to the rarity of IgG4-RD, the exact prevalence and incidence of IgG4-RH is not well known nor reported; and as such the prevalence of pituitary gland involvement is also yet to be determined^29^. Moreover, diagnosing IgG4-RH remains challenging, often resulting in it being reported as idiopathic hypophysitis^29^. IgG4-RH can manifest as either pituitary dysfunction leading to hypopituitarism or mass effect causing headaches^30,31^. Visual disturbances have also been reported, the etiology is often due to compression of the optic chiasm by the enlarged pituitary gland, which causes a decrease in the visual acuity, color perception and visual field defects^32,33^.

Hypophysitis can be classified based on the anatomic location of the pituitary involvement, the histological appearance and the etiology. Anatomically hypophysitis is classified into adenohypophysitis, infundibuloneurohypophysitis and Panhypophysitis ^34^. The clinical findings in IgG4-RH are quite similar to other causes of hypophysitis and include headaches, visual defects, fatigue and isolated or multiple hormone deficiency due to adenohypophysitis^35^. Infundibuloneurohypophysitis causes lymphocytic infiltration of the infundibulum, stalk and the posterior lobe of the pituitary gland can lead to Diabetes Insipidus (DI)^36^. The etiological classification distinguishes between primary and secondary hypophysitis, with primary hypophysitis being idiopathic with an autoimmune pathogenesis like in IgG4-RH^34^. Secondary hypophysitis refers to cases where a causative agent has been identified for instance immune checkpoint inhibitors such as CTLA-4 inhibitors^37,38^.

IgG4-RH manifestations are dependent on the specific hormonal deficiencies leading to a variety of symptoms from polyuria, polydipsia, erectile dysfunction, malaise and muscle weakness^28,39^. IgG4-RH doesn’t follow a consistent hypopituitarism pattern and more commonly causes panhypopituitarism^40^. Among isolated hormone deficiencies, ACTH deficiency is the most widespread form of hypopituitarism causing nonspecific symptoms such as fatigue weakness, anorexia, weight loss, nausea, vomiting and hypotension and if left untreated it can lead to a severe adrenal crisis^31^ .Gonadotropin deficiency is the second most common form, and it can cause gynecomastia, infertility and lack of libido ^31,41^. Followed by secondary hypothyroidism with a clinical picture of cold intolerance, lethargy, weight gain, etc^31,42^.

Symptoms due to hormonal deficiencies are rarely the only manifestation of IgG4-RH, a registry-based, retrospective cohort study published by Chakraborty et al found that headaches were the most common complaint, followed by menstrual abnormalities reported in the female patients^29^. In addition, patients also exhibited extracranial symptoms due to IgG4-RD including retroperitoneal fibrosis leading to ureteric obstruction and hydronephrosis, pancreatitis, adamantinomas, craniopharyngioma, primary testicular failure and pelvic adhesion^29^. Bhargava et all reported that in their retrospective cohort that TSH deficiency was the most common hormone deficiency followed by deficiency of ACTH then gonadotropin^43^. Signs of hypophysitis and pituitary enlargement were reported in the scans of these patients with extension into the suprasellar space and thickening of the pituitary stalk^43^.

If the pituitary gland is the sole organ involved, a diagnosis of IgG4-RD based on the 2019 ACR/EULAR classification criteria cannot be established^44,45^. The diagnosis of IgG4-RD is still possible without confirmatory histopathological evidence provided that the patient has: histologic findings of the pituitary gland with a lymphoplasmacytic infiltrate, MRI findings consistent with hypophysitis (presence of a sellar mass or thickening of the pituitary stalk), biopsy of another affected organ demonstrating IgG4-RD involvement, elevated IgG4 serum level (>140 mg/dL) or a therapeutic response to glucocorticoid evidenced by reduction of the pituitary mass and improvement of symptoms^34^.

As mentioned previously, establishing the diagnosis of IgG4-RH is quite challenging, Leporati et al adopted an algorithm for management and diagnosis of IgG4-RH; serum IgG4 levels must be measured in all patients with clinical suspicion of hypopysitis and the presence of a sellar mass on imaging^34^. If serum IgG4 is greater than 140 mg/dl glucocorticoid treatment should be initiated with referral to a multidisciplinary team if accessible, if the symptoms resolve after treatment initiation, then IgG4-RH is confirmed, and the patient should be followed up yearly with endocrinological testing and monitoring of pituitary hormone levels^34^. Biopsy should be obtained if glucocorticoid therapy fails to resolve the symptoms, then IgG4 diagnosis is made if the specimen was positive for features suggestive of IgG4 involvement^43^. If there are signs of multi-organ involvement on systemic imaging, biopsy of the affected organ should be made, and diagnosis is confirmed with the presence of characteristic pathological features^34^.

The primary approach in treatment of IgG4-RH heavily relies on steroids the starting dose is 30-40 mg of prednisone^46^. In cases of poor responsiveness, patients might be switched to azathioprine or rituximab, to date no studies have compared the efficacy of one over the other for the treatment of IgG4-RH^46,47^. Certain studies have suggested initiating treatment with dopamine agonists such as bromocriptine to treat autoimmune hypophysitis given the potential immunomodulatory effect of prolactin, though effectiveness of this approach is uncertain and has not been established in IgG4-RH^46,48^ Surgery is now reserved for patients suffering from significant visual deficits, reduced visual acuity, oculomotor nerve palsies or resistance to medical therapy a key advantage of surgery is obtaining tissue for histopathological analysis ensuring a definitive diagnosis^46^. Radiotherapy might also be considered if other options failed or in the case of recurrence, focused radiotherapy has been used as a potential therapeutic approach for managing lymphocytic hypophysitis, but no studies have reported its use for IgG4-RH^46,49^.

## 4. Thyroid Involvement

Thyroid involvement in IgG4-RD can be categorized into four main forms: IgG4-related Hashimoto's thyroiditis, a distinct fibrosing variant of Hashimoto's thyroiditis, Riedel's thyroiditis, and Graves’ disease with elevated IgG4 levels^50,51^.

### 4.1 Riedel’s thyroiditis

Riedel's thyroiditis (RT) is also called invasive fibrous thyroiditis^52^. RT is relatively uncommon and is a characteristic entity of the IgG4-RD spectrum, often occurring in patients who either have a previous diagnosis of IgG4-RD or are subsequently discovered to have fibrosis other organs, such as the retroperitoneum, pancreas, or mediastinum^53,54^. Out of 56,700 thyroidectomies done at Mayo Clinic between 1920 and 1948, Riedel’s thyroiditis represented only 37 cases^55^. In RT, the normal thyroid tissue is widely or partially replaced by fibrous tissue, severe distortion of the thyroid follicular architecture, obliterative phlebitis, and an immune infiltration of lymphocytes, plasma cells, and eosinophils are also affecting the thyroid tissue^54^. The fibrosis is not limited to thyroid tissue but also extends to nearby tissues, causing compressive symptoms and so a thorough evaluation is needed to determine the extent of the fibrosis to nearby structures, including the trachea, esophagus, and recurrent laryngeal nerves^52,54^. Due to the firm and fibrous texture of the thyroid gland in RT, it can be misdiagnosed as malignancy, raising concerns about anaplastic thyroid cancer, thyroid angiosarcoma, or lymphoma^56,57^.

Regarding diagnosis and treatment, most patients with RT initially present in a euthyroid phase; however, as the disease progresses, approximately 40% develop hypothyroidism^8^. Definitive diagnosis requires ultrasound-guided needle biopsy or open surgical biopsy, revealing dense collagen fibers that resemble keloid bands, along with marked lymphocytes and plasma cell infiltration^54,58^. Thyroid ultrasound (+/-doppler) and elastography can complement the diagnosis, on sonography hypo-echoic hypo-vascular mass extending to the surrounding tissues involving the carotid arteries^54,59^. Elastography would demonstrate stiff, inflamed, fibrotic tissues that is used is neck ultrasound along with Doppler or elastography for a more comprehensive approach^26,28^. In RD, Neck ultrasonography may reveal a hypo-echoic hypo-vascular mass extending to the surrounding tissues involving the carotid arteries. Elastography would demonstrate stiff, inflamed, fibrotic tissues^60^.

The main treatment option for RT is glucocorticoids^52^. Given it’s role in decreasing fibroblast activity, tamoxifen has been used in the past and studies have shown that it can be considered as an adjunctive therapy along with corticosteroids^61,62^. A study by Few et al evaluated 4 patients with biopsy confirmed RT before and after receiving tamoxifen, over an observation period of 1 to 4 years, all patients subjective improvement, while objective disease resolution was observed in 50% to 100% of the cases^62^. Rituximab has also been used with patients experiencing unresponsiveness to glucocorticoids and tamoxifen^61^. Thyroidectomy is indicated in cases whenever the patient presents with any signs of significant cervical compression, for cosmetic reasons, and lastly, when the cytology of the thyroid nodule appears cancerous^63^.

### 4.2 Hashimoto’s thyroiditis

In contrast to RT, Hashimoto’s thyroiditis (HT) is much more common and typically presents differently. In developing nations, HT is the primary cause of hypothyroidism, affecting 0.1% to 2% of the population and in contrast to IgG4-RD, it’s tenfold more common in women compared to men^64,65^.HT can be subdivided into two categories: IgG4 thyroiditis and non-IgG4 thyroiditis; compared to non-IgG4 thyroiditis, IgG4-related Hashimoto thyroiditis deteriorates faster and is associated with elevated levels of antithyroglobulin and antithyroperoxidase and commonly presents as subclinical hypothyroidism^8,51^. Unlike RT, IgG4-related Hashimoto thyroiditis is not linked to any systemic features of IgG4-RD, as in RT, which can be useful to distinguish between the two diseases^51^.

Another variant, the fibrosing variant of HT is marked by a firm, enlarged, and lobulated thyroid affecting one-third of thyroid parenchyma, and it is presented only in 10% of the cases^8,66,67^. The fibrosis in this variant is characterized by dense keloid-like bands that convert thyroid architecture into a lobulated gland^67^. Clinically and radiologically the fibrosing variant can resemble RT because of the rapid growth that is present in both conditions and resembling imaging features. However, unlike RT, which causes destruction to nearby structures, fibrosis in HT is limited to thyroid tissues only, which serves as another differentiating feature^51,66,67^. Additionally, contrasting from both RT and the fibrosing variant of HT, the IgG4-related HT variant exhibits more hypoechogenicity on ultrasound^68^.

In HT associated with IgG4-RD, ultrasound is characterized by reduced echogenicity due to destruction of the thyroid follicles and immune cell infiltration, making the tissue resemble adjacent muscles ^67^. On the other hand, the fibrous variant is associated with irregular and nodular patterns caused by the extensive disposition of collagen fibers^63,67^.

In addition to thyroid hormone replacement, administration of a brief glucocorticoid regimen should be considered in patients with the IgG4-RD variant of HT to reduce the inflammation and decrease the risk of permanent hypothyroidism^67^.

In contrast to thyroid involvement in IgG4-RD, the parathyroid is rarely affected. Parathyroid adenomas with marked lymphocytic infiltration associated with IgG4-RD have been reported a handful of times, and to date, only 11 cases have been reported^69^. Aspiration cytology of these lesions are often suggestive of papillary thyroid carcinoma and after resection of the specimen, a diagnosis of parathyroid adenoma is noticed with parathyroid cell hyperplasia and nuclear atypia embedded in a fibrotic stroma with prominent lymphocyte infiltration comprising a germinal center^69^. Other associated histological features indicated a strong association with IgG4-related diseases, such as marked elevation of IgG4-positive plasma cells, IgG4/IgG-positive plasma cell ratio, and storiform fibrosis and lastly, obliterative phlebitis^69^.

## **5.** Pancreatic involvement

Pancreatic involvement is common in IgG4-related disease, either as AIP or lymphoplasmacytic sclerosing^11,70,71^. AIP is further subdivided into type 1 and type 2 with type 1 being the form that is most frequently associated with IgG4-RD, type 2 AIP is also called idiopathic autoimmune pancreatitis, histologically it differs from type 1 AIP by the presence of features resembling granulocytic epithelial infiltration into the pancreatic duct epithelial layer^72,73^. Pathologic features of type 1 AIP include lymphoplasmacytic infiltration and fibrosis around pancreatic ducts^11^. At a tissue level, The inflammation and fibrosis affects the pancreatic ducts^74^.

The primary clinical manifestations in individuals with autoimmune pancreatitis include jaundice, abdominal discomfort, weight loss and newly developed diabetes mellitus^11^. These symptoms may be relatively unspecific and occur in cases of pancreatic cancer, making diagnosis challenging, imaging modalities such as CT scan, MRI and endoscopic ultrasound (EUS) along with blood tests for markers like IgG4 and CA 19-9, are essential for distinguishing autoimmune pancreatitis from pancreatic malignancy^71,75^. In addition, an improvement in symptoms after corticosteroids further supports AIP diagnosis^71^.

AIP can also impair the endocrine functions of the pancreas affecting its insulin secretion, especially in advanced or fibrotic stages of the disease^11,74^. According to various studies, endocrine dysfunction (mostly manifesting as new onset diabetes) is seen in upwards of 60% of patients with AIP^72,76^. The inflammatory infiltrate and fibrotic process initiated in the pancreas as a direct effect of IgG4 damages its exocrine & endocrine cells which differentiates IgG4-RD from classic type 1 diabetes. Moreover, the immune system especially CD8+ T-cells aims to target the islets, which houses the insulin-producing beta cells^74^. This immune attack automatically leads to a decreased functioning of the beta cells, and to the rise in blood sugar levels^74^. The consequence of endocrine dysfunction is reduced insulin producing capacity which eventually leads to diabetes^77^. Less commonly, patients with AIP present with exocrine function impairments such as steatorrhea, fatty stool, indicating reduced exocrine enzyme production and post prandial distention, a sense of exaggerated fullness after meals, reflecting malabsorption secondary to exocrine insufficiency^78^.

Diabetes linked to autoimmune pancreatitis (AIP), can appear at any point during the illness, but in more than half of cases, it’s already present when AIP is first diagnosed^74^. The severity of disease varies amongst individuals ranging from mild disease requiring oral medications or life style modifications to insulin dependent disease^79^. Paradoxically, glycemic control improves in patients with AIP with corticosteroid treatment This type of diabetes usually resolves with corticosteroid treatment, about 60% of individuals with AIP related diabetes have shown positive prognosis in the short and long term^72,74,76^. In terms of reversibility, the disease proves to be reversible with appropriate management with glucocorticoid therapy. It is the first line therapy for IgG4 pancreatitis due to its effective glycemic control. According to short term studies, in 55-66% of patients with the simultaneous onset of AIP & diabetes there have been reports of improvement. On the other hand, in patients with preexisting diabetes, 36-54% of the cases have been repeated to improve following therapy which is reflected by reduced insulin requirements or lower HbA1c levels. Complete reversibility is not guaranteed based on long term follow up studies yet they have demonstrated improvement in 63% of patents following therapy ^80^.

In addition to immunosuppression, more severe or advanced cases with markedly impaired glycemic control may require insulin therapy, especially when irreversible damage or shrinking of the pancreas has occurred^72,76^. The outcome generally depends on how severe the pancreas is affected^12,74^. There are several predictors of endocrine recovery and outcome which are aspirated with higher remission and fewer relapse rates including early steroid therapy initiation and radiologic findings following therapy^81^. Better outcomes are expected when pancreatic swelling resolves following therapy whereas worse outcomes associated with higher relapse rates and poorer endocrine function is expected when pancreatic swelling persists or it progresses into atrophy^81,82^. Additionally, serological tests in coding IgG4 levels reflect recovery. By which persistently elevated IgG4 levels despite ongoing therapy is associated with increased relapse rates^83^.However, the pancreas seems to self-repair: some ductal cells start to express insulin and glucagon, hinting that they might be trying to become new islet cells^74^. These regenerating ductal cells show high levels of a protein called IPF-1, a key factor in beta-cell development which leads to a final analysis that the pancreas usually continue to regenerate its insulin-producing capacity^74^.

As mentioned earlier, corticosteroids treatments are being used to treat wide ranges of diseases, including AIP. Despite that, it has linked to many side effects such as new onset of diabetes in people with already diagnosed disease, the latter is called “steroids-induced hyperglycemia” or in people with no pre-existing disease, ”steroids-induced diabetes”^84^. Additionally, it can lead to prolonged stays in hospitals, increased risks of infections and with transplant patient, Graft dysfunction^85^. To avoid this, screening should be considered in these groups of patients along with initiation of insulin therapy^85,86^.

## 6. Diagnostic Workup in Suspected Endocrine IgG4-RD

**Figure 2:**
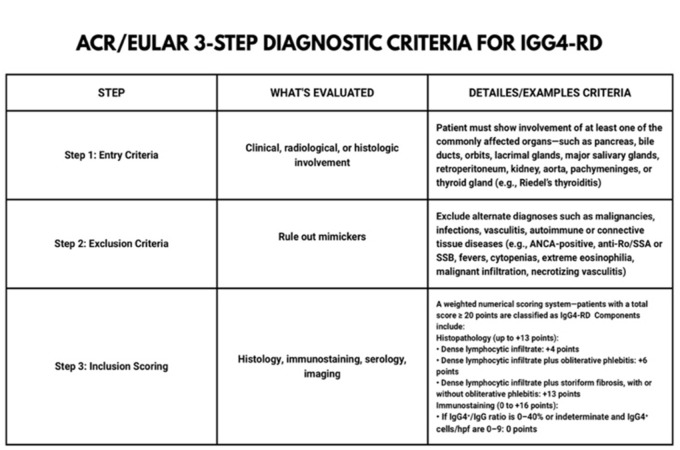
The three step classification criteria proposed by the American College of Rheumatology (ACR) and European League Against Rheumatism (EULAR)

The diagnosis of IgG4-RD poses significant challenges due to its nonspecific symptoms and diverse organ involvement^26^. Since treatment depends on precise and prompt diagnosis, addressing these challenges is crucial. The American College of Rheumatology (ACR) and European League Against Rheumatism (EULAR) propose 3-step classification criteria that has been widely used (**Figure 2**). This involves evaluation of serological tests, clinical signs, histopathology, and imaging^44^. The first step is the inclusion criterion which refers to the clinical or radiologic involvement or the presence of a pathologic inflammatory lymphoplasmacytic infiltration in at least one organ out of the usual 11 (pancreas, biliary tree, salivary glands, orbits, kidney, lungs, aorta, retroperitoneum, pachymeninges, and thyroid as Reidel’s thyroiditis)^44^. The second step is the exclusion criteria which are determined by the presence of 32 features that if present rule out an IgG4 diagnosis, these include clinical (fever, no response to glucocorticoids), serologic (leukopenia, unexplainable thrombocytopenia with no explanation, peripheral eosinophilia, positive autoantibodies that can be attributed to a connective tissue disease), radiologic (radiologic findings suspicious for malignancy or infection, rapid radiologic progression, long bone abnormalities consistent with Erdheim-Chester disease, splenomegaly) and pathologic (cellular infiltrates suggesting, markers consistent with inflammatory myofibroblastic tumour, prominent neutrophilic inflammation, necrotizing vasculitis, prominent necrosis, primarily granulomatous inflammation, pathologic features of macrophage/histiocytic disorder), and a known diagnosis of the following: Multicentric Castleman’s disease, Crohn’s disease or ulcerative colitis (if only pancreatobiliary disease is present), Hashimoto thyroiditis (if only the thyroid is affected) as part of the 32 features^44^. The final step, weighted inclusion scoring system, only proceeds if the presenting case meets the inclusion requirements and not the exclusion which is summed up as a preliminary score of ≥ 20 points that serves as the threshold for meeting the IgG4-AZ classification criteria (specificity 97.8%, sensitivity 82.0%)^44^.The three step classification criteria proposed by the American College of Rheumatology (ACR) and European League Against Rheumatism (EULAR) is showed in **Figure 2**.

Detection of elevated plasma IgG4 levels alone was initially considered sufficient to support an IgG4-RD diagnosis, however, the current understanding is that showed that serum IgG4 lacks specificity and sensitivity, making it inadequate as a sole diagnostic criterion and approximately 30% of diagnosed patients have IgG4 levels within the normal range^87,88^. Elevated IgG4 levels (especially when >135 mg/dL), when accompanied by typical multi-organ involvement and clinical features, further support the diagnosis of IgG4-RD^87^. Additionally, IgG4 serum levels are established markers of disease activity, with elevations linked to aggressive progression, multi-organ involvement, inflammatory marker changes, low complement levels, and poor treatment response^89-91^.

Laboratory tests often show nonspecific findings such as, elevations in ESR because of hypergammaglobulinema in untreated disease, less commonly moderately elevated inflammation markers (CRP in 25% of cases) reflecting an acute phase reactant discordance (high ESR and low CRP) and mild eosinophilia^92^. The diagnostic potential of serum and tissue levels of IgG4 and IgG2 has been noted, particularly when considering the ratio of IgG4-positive to IgG-positive plasma cells^92^. Certain markers such as laminin 511-e8, galectin-3, prohibitin and annexin A11 have been implicated in the pathophysiology of IgG4-RD and can be used as potential biomarkers in the future^92,93^ .

Biopsy and histopathology of affected organs remain the gold standard for diagnosing IgG4-RD by identifying IgG4+ cells and an IgG4/IgG ratio > 0.4^51^. Diagnostic criteria require biopsy findings of solid lymphoplasmacytic infiltrates, storiform fibrosis, and obliterating phlebitis^51,71^. Less characteristic features include non-obliterating phlebitis, obliterating arteritis, and eosinophilic infiltration^94^. Confirming diagnosis requires immunostaining for IgG4+ and IgG+ plasma cells, quantified per high-power field and by the IgG4+/IgG+ plasma cell ratio^87,95^. In addition to directly visualizing the storiform pattern of fibrosis in biopsy specimens of affected organs, the use of cellular markers of inflammatory fibrosis could support IgG4-RD diagnosis, as fibrous inflammation results from immune-mediated IgG4 organ infiltration^96^. Fibrosis markers include serum CC-chemokine 18 (CCL18), correlating with disease severity and organ involvement; tissue infiltration by M2 macrophages and their cytokines (IL-33, IL-10, CCL18); and fibrosis extent^96,97^. Serum plasmablast levels rise after organ-specific fibroinflammatory changes, showing 95% sensitivity and 82% specificity, and predict prognosis post-treatment^16,98^. Imaging is not confirmatory but helps with differential diagnosis and assessing disease extent before obtaining biopsies^91^. Advanced diagnostic tools include FDG PET/CT (18F-fluorodeoxyglucose positron emission tomography) and 68Ga-FAPI PET/CT (fibroblast activator protein inhibitor labeled with gallium-68). FDG PET/CT effectively estimates disease severity by highlighting active inflammatory lesions, staging and monitoring progression, assessing treatment response, and guiding biopsies^99,100^. 68Ga-FAPI PET/CT shows potential as a more detailed imaging option^101,102^.

Suspected endocrine manifestations of IgG4-RD necessitate specific hormonal and functional testing for accurate diagnosis and treatment due to its significant influence on clinical outcomes^34^. Endocrine organ involvement includes pituitary (most frequently involved), thyroid, and pancreatic manifestations while adrenal manifestations are mostly relegated to sparse case reports^103^. IgG4-related hypophysitis reflects pituitary gland involvement which may present as an isolated hormone deficiency or panhypopituitarism and thus, basal pituitary hormonal testing is needed such as measurements of ACTH, cortisol, TSH, free T4, LH, FSH, prolactin, GH, and IGF-1 alongside ACTH stimulation or insulin tolerance test as part of dynamic testing^34^. On the other hand, TSH levels, free T4, and anti-thyroid antibodies, alongside imaging and biopsy when indicated, are performed when evaluating for Riedel’s thyroiditis or chronic autoimmune thyroiditis with fibrosis during thyroid involvement. In conclusion, diagnosing IgG4-RD remains challenging due to its diverse clinical presentations, overlap with other conditions, and diagnostic limitations. Since no single test is definitive, an integrated approach is essential. Ongoing research promises improvements in early diagnosis, disease monitoring, and treatment evaluation. Addressing current diagnostic challenges is key to achieving optimal patient outcomes.

## 7. Treatment Strategies and Endocrine Outcomes

Therapeutic intervention in IgG4-related disease primarily aims to suppress disease progression and avert permanent structural damage to affected organs^104^. Treatment efficacy is influenced by the disease duration, degree of fibrosis, and specific organ involved, glucocorticoids remain the gold standard treatment in IgG4-RD due to extensive evidence supporting their clinical efficacy^89,104^. The starting dose typically ranges from 0.5-1.0 mg/kg/day, modified based on disease activity, and the dose generally maintained for a period of 2 to 4 months or until inducing effective disease remission^104^. Most patients exhibit favorable responses to appropriate therapy, involving regression of lesions and normalization of laboratory parameters, even in the presence of irreversible organ damage^44^. It is important to highlight that a delay in starting steroids after symptom onset or having retroperitoneal fibrosis (RF) at the time of diagnosis are two principal risk factors considered to contribute significantly to the likelihood of relapse^105,106^. Additional prognostic factors that are extensively linked to higher risk of relapse in patients with IgG4-RD are high serum IgG4 concentrations at the time of diagnosis and systemic involvement^107,108^.

The use of immunosuppressants can potentially lower the need for higher doses of steroids thereby contributing to lower relapse rate^107^. Azathioprine has been used alongside corticosteroids to induce remission however, most patients eventually required alternative therapy, and , transitioning from glucocorticoids to azathioprine in active IgG4-related pancreatitis often fails highlighting its limited-long term effectiveness^72^. Yunyun et al reported that the addition of mycophenolate to glucocorticoids reduced relapse risk significantly more than glucocorticoids alone by 21%, however like azathioprine, MMF is not invariably sufficient to maintain remission, especially in severe or multi-organ disease^109^. In a case series of 10 patients, Della-Torre et al reported that the methotrexate was able to maintain remission after the use of corticosteroids in 60% of cases while the others demonstrated partial remission^110^.

One of the most effective treatments for IgG4-RD is rituximab, the CD20 receptor is only expressed on B cells and not on plasma cells^105^. Since plasma cells are considered the main source of immunoglobulin G (IgG) antibodies production, the use of rituximab as a treatment for IgG4-RD might sound counterintuitive however, rituximab has been clinically proven to significantly lower the amount of circulating IgG levels and improve the associated symptoms^87,105,111^. Plasma cells are essentially classified into short-lived plasma cells depending on the continuous replenishment from CD20+ B cells, and long-lived plasma cells, short lived plasma cells are the primary producers of large amounts of pathogenic antibodies in autoimmune disorders^2^. Rituximab reduces the levels of disease-related antibodies by depleting the B cell precursors, thereby decreasing their counts rituximab has been found to reduce relapse rates and as such is especially useful in cases where patients experience poor response and intolerable adverse effects to glucocorticoids and other steroid-sparing agents^112^.

Hormonal replacement is generally required in cases of IgG4-related hypophysitis with glucocorticoids for secondary adrenal insufficiency, levothyroxine for central hypothyroidism and desmopressin if central diabetes insipidus develops due to posterior pituitary involvement^28^. As discussed earlier, corticosteroids alone are often enough to improve the symptoms in IgG4-related thyroiditis, however if hypothyroidism develops, thyroid hormone replacement can be utilized^64^. The long-term outcomes in IgG4-RD differ by the affected gland, for instance most patients with IgG4-RH suffer from panhypophysitis or anterior hyopopituitarism following surgical resection^44^. The prognosis of autoimmune pancreatitis is uncertain due to factors such as disease relapse, persistent exocrine and endocrine insufficiency, and the associated malignancies like pancreatic cancer^11^. Riedel’s thyroiditis generally has favorable survival but significant comorbidity^55^. Since the disease progresses through extensive fibrosis, compressive symptoms often occur, as well as hypothyroidism due to destruction of the thyroid tissue^55^. As it falls into the IgG4-RD spectrum patients might develop multifocal fibrosclerosis with multisystem involvement^55^.

## 8. Conclusion

Endocrinological manifestations of IgG4-related disease remain an underdiagnosed but significant component of this multisystemic fibroinflammatory disease. Irreversible hormonal dysfunction and glandular fibrosis can arise if not promptly diagnosed and managed. Despite glucocorticoids being the mainstay of treatment, long term follow-up and individualized treatment plans remain crucial due to the risk of relapse and chronic disease. Most importantly, the current base of evidence is limited by a predominance of retrospective cohort studies, case reports, and small case series. Multidisciplinary approaches and greater clinical awareness are required for prompt diagnosis, characterizing the disease spectrum, and providing the best standard of care for patients with endocrinological manifestations of IgG4-related disease. Future research studies should focus on identifying reliable biomarkers that are predicitve of endocrine recovery and relapse as well as evaluate steroid sparing treatments in a standardized manner.

## Bullet points

- **IgG4-related is a distinct fibroinflammatory condition characterized by tumefactive lesions, storiform fibrosis, and dense infiltration of IgG4-positive plasma cells across multiple organs.**

- **Diagnosis by elevated serum IgG4 levels help differentiate IgG4-related disease from other malignancies and autoimmune conditions.**

- **Glucocorticoids have shown great efficacy in managing the disease while other immunobiological treatments targeting B cells such as rituximab have been shown to maintain remission and refractory cases.**

- **How genetic and environmental factors contribute to the different presentations and organ tropism is still unknown.**

- **It is still controversial if earlier diagnosis and treatment prevent irreversible fibrosis and organ dysfunction in IgG4-related disease patients.**

## Publisher’s note 

All claims expressed in this article are solely those of the authors and do not necessarily represent those of their affiliated organizations, or those of the publisher, the editors and the reviewers. Any product that may be evaluated in this article, or claim that may be made by its manufacturer, is not guaranteed or endorsed by the publisher.
